# In Silico Description of the Direct Inhibition Mechanism of Endothelial Lipase by ANGPTL3

**DOI:** 10.3390/ijms25063555

**Published:** 2024-03-21

**Authors:** Linda Montavoci, Omar Ben Mariem, Simona Saporiti, Tommaso Laurenzi, Luca Palazzolo, Alice Federica Ossoli, Uliano Guerrini, Laura Calabresi, Ivano Eberini

**Affiliations:** 1 Dipartimento di Scienze Farmacologiche e Biomolecolari, Università degli Studi di Milano, Via Giuseppe Balzaretti 9, 20133 Milan, Italy; linda.montavoci@unimi.it (L.M.); omar.benmariem@unimi.it (O.B.M.); tommaso.laurenzi@unimi.it (T.L.); luca.palazzolo@unimi.it (L.P.); uliano.guerrini@unimi.it (U.G.); 2 Analytical Excellence and Program Management, Merck Serono S.p.A., Via Luigi Einaudi 11, Guidonia Montecelio, 00012 Rome, Italy; simona.saporiti@merckgroup.com; 3 Centro E. Grossi Paoletti, Dipartimento di Scienze Farmacologiche e Biomolecolari, Università degli Studi di Milano, Via Giuseppe Balzaretti 9, 20133 Milan, Italy; alice.ossoli@unimi.it (A.F.O.); laura.calabresi@unimi.it (L.C.); 4 Data Science Research Center (DSRC), Università degli Studi di Milano, Via Giuseppe Balzaretti 9, 20133 Milan, Italy

**Keywords:** ANGPTL3, endothelial lipase, molecular dynamics, protein–protein docking, HDL remodeling, lipid metabolism

## Abstract

Angiopoietin-like protein 3 (ANGPTL3) is a plasmatic protein that plays a crucial role in lipoprotein metabolism by inhibiting the lipoprotein lipase (LPL) and the endothelial lipase (EL) responsible for the hydrolysis of phospholipids on high-density lipoprotein (HDL). Interest in developing new pharmacological therapies aimed at inhibiting ANGPTL3 has been growing due to the hypolipidemic and antiatherogenic profile observed in its absence. The goal of this study was the in silico characterization of the interaction between ANGPTL3 and EL. Because of the lack of any structural information on both the trimeric coiled-coil N-terminal domain of ANGPTL3 and the EL homodimer as well as data regarding their interactions, the first step was to obtain the three-dimensional model of these two proteins. The models were then refined via molecular dynamics (MD) simulations and used to investigate the interaction mechanism. The analysis of interactions in different docking poses and their refinement via MD allowed the identification of three specific glutamates of ANGPTL3 that recognize a positively charged patch on the surface of EL. These ANGPTL3 key residues, i.e., Glu154, Glu157, and Glu160, could form a putative molecular recognition site for EL. This study paves the way for future investigations aimed at confirming the recognition site and at designing novel inhibitors of ANGPTL3.

## 1. Introduction

Angiopoietin-like proteins (ANGPTLs) are structurally related to the angiopoietins family, which includes eight members (ANGPTL 1–8). These proteins are involved in different biological processes, such as lipid metabolism, atherosclerosis, and cancer [1]. At the structural level, they share common features such as an N-terminal ‘coiled-coil domain’ (CCD) responsible for oligomerization, a linker region, and a C-terminal fibrinogen-like domain (FLD) that binds Tie2 receptors [2].

Human ANGPTL3 (UniProtKB ID: Q9Y5C1) is composed of 460 amino acids, in addition to a signal peptide of 16 amino acids necessary for secretion [3], resulting in a molecular weight of approx. 70 kDa, and it is mainly expressed in the liver [4]. Experimental evidence suggests that ANGPTL3 can oligomerize, forming both trimers and hexamers, and it is more stable as a trimer [5]. The structural analysis of ANGPTL3 trimers reveals elongated structural envelopes with a length consistent with other coiled-coil proteins of a similar size [5]. The linker region between N-terminal and C-terminal domains is cleaved by furin and paired basic amino acid-cleaving enzyme (PACE4) [6] in the sites between Arg221-Ala222 and Arg224-Thr225 [4]. The order of these cleavage events remains unclear, as the full-length protein is present in plasma [4]. After the cleavage, the C-terminal portion participates in the angiogenesis pathway, while the N-terminal part is involved in lipid metabolism [4], more specifically in high-density lipoprotein (HDL) remodeling and in very low-density lipoprotein (VLDL) metabolism. Among the domains of ANGPTL3, only the 3D structure of the fibrinogen-like domain has been experimentally solved by crystallography as a globular trimer (PDB ID: 6EUA [7]), while structural information for the other regions is missing.

Experimental studies have demonstrated that ANGPTL3 inhibits lipoprotein lipase (LPL) [8] and endothelial lipase (EL) (UniProtKB ID: Q9Y5X9) [9,10]. LPL hydrolyzes triglycerides on VLDL and chylomicrons, releasing free fatty acids and monoacylglycerols, forming smaller and less lipid-rich VLDL [11]. EL hydrolyzes phospholipids on mature α-HDL, producing discoidal preβ-HDL, a process at the basis of HDL remodeling. While the role of ANGPTL3 in apoB-containing lipoprotein metabolism and its inhibition mechanism of LPL have already been characterized, its role in HDL metabolism and the interaction mechanism with EL have not been fully clarified as yet. From a structural perspective, EL presents a homodimeric head-to-tail conformation in which each monomer, formed by 500 amino acids, is organized in two domains: the ‘lipase domain’ (Val49-Ala340), with a catalytic function, and the ‘PLAT-LH2 domain’ (Tyr347-Arg488), critical for the interaction with membranes and lipids. Actually, EL can be found either bound to membranes or in the bloodstream, but recently it has been reported that EL is inhibited to a lower extent by ANGPTL3 when bound to a membrane [12]. The mechanism of inhibition of EL by ANGPTL3 is still not known, but it has been demonstrated that it is direct and different from that observed for LPL [13]. Another study demonstrated that the N-terminal domain of ANGPTL3 reduces EL activity in a similar way to the full-length protein, indicating that the N-terminal domain is sufficient to inhibit EL [10].

The structural mechanism behind the inhibition of EL promoted by ANGPTL3 is worth investigating, as it has been observed that subjects with a loss-of-function mutation in the ANGPTL3 gene show a peculiar phenotypic lipid profile, named familial combined hypolipidemia (FHBL2, OMIN #605019) [14]. This clinical condition is characterized by low plasma levels of total cholesterol (TC), triglycerides (TG), VLDL-C, LDL-C, HDL-C, ApoB, ApoA-I, and free fatty acids, due to the lack of inhibition of LPL and EL. FHBL2 carriers not only do not show increased cardiovascular risk [15], but, on the contrary, their lipid profile makes them less prone to undergo atherogenic risk [14]. According to the lower levels of pro-atherogenic lipoprotein in these patients, ANGPTL3 can be considered as a promising therapeutic target in the treatment of dyslipidemia. However, at the moment, only one monoclonal antibody, Evinacumab, which recognizes and blocks ANGPTL3, is commercially available and is used for the treatment of familial hypercholesterolemia [16].

Given this background, the aim of this work was to model for the first time the 3D structure of both the ANGPTL3 N-terminal domain and EL and to investigate their interaction at an atomistic level via computational methods. To this purpose, a combination of homology modeling and de novo strategies, protein–protein docking, and molecular dynamics (MD) simulations was used to shed light on a putative molecular recognition mechanism between these two proteins.

## 2. Results

### 2.1. Ab Initio Modeling of the N-Terminal Domain of ANGPTL3

The ANGPTL3 secondary structure was predicted by PsiPred, in agreement with the existing literature; the results showed the presence of a long α-helix at the N-terminal domain interspersed with short loops (Supplementary Figure S1). Additionally, most papers in the literature, as well as available experimental structures of the C-terminal of ANGPTL3, suggest that this protein oligomerizes, forming a homotrimer in its functional form. Therefore, the software CCBuilder 2.0 was used, generating an ideal trimeric coiled-coil structure. This software generates “ideal” structures that cannot be considered reliable models without further processing. Additionally, because of the features of the α-helices, the starting residue would determine the whole winding. Therefore, as no specific information was available, in order to obtain different exposed residues and to monitor their behavior in solution, four ANGPTL3 models were generated, each starting from a different consecutive residue (18 to 21), to observe different exposed residues and their behavior in solution. All models were then submitted to 100 ns long MD simulations to allow for adjustments of the ϕ and ψ main chain dihedral angles, which CCBuilder had set to “ideal” values (Supplementary Figure S2). In order to identify the best model to use for further analysis, the secondary structure content of the four obtained structures was analyzed during the MD trajectory; a large prevalence of residues in the α-helix was observed, with the notable exception of the N- and C- terminals and, more interestingly, of the region between Pro153 and Glu160. This finding is coherent with the presence of three prolines in this region, whose cyclic side chain hinders helicity, and it is also in full agreement with the PsiPred prediction (Supplementary Figure S3). 

In addition, both the overall root-mean-square deviation (RMSD) and the root-mean-square fluctuation (RMSF) were computed for the four models, showing very similar trends and no plateau state for the RMSD within the simulated time (Supplementary Figure S4). However, comparing the RMSF profiles (Supplementary Figure S5), only ANGPTL3 CC 19 showed a significant increase in fluctuations, suggesting that this is the least stable structure. Cluster analysis on the four trajectories is reported in Supplementary Figure S6, where ANGPTL3 CC 18 appears to be the first to approach a dynamic equilibrium state, moving from its initial “ideal” coiled-coil set-up to more realistic conformations, with backbones bending to a different extent around residues 135–136.

The Ramachandran plots calculated on the medoids of the most populated clusters show that most of the points are in allowed areas (Supplementary Figure S7), indicating that in all the models, the backbone dihedral angles were correctly relaxed.

**Figure 1:**
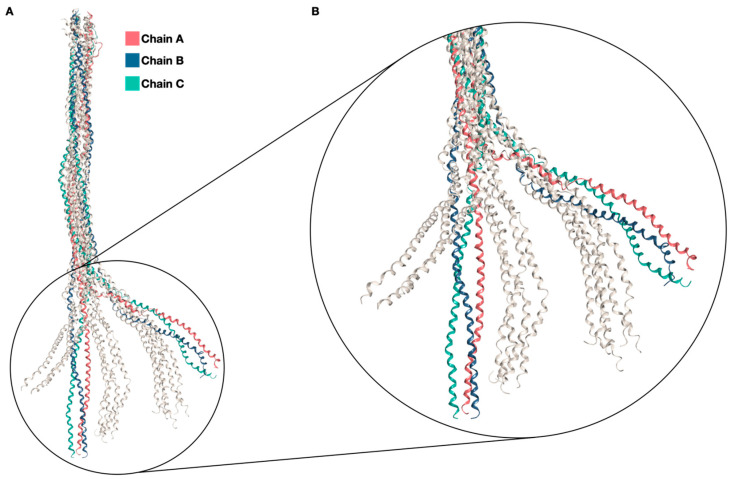
ANGPTL3 structures. (**A**) Significant conformational changes can be observed, starting from the initial “ideal” coiled-coil structure generated by CCBuilder (colored, on the left of the image and the magnification), which can then assume a range of more realistic conformations bending, to a varying extent, at ~Gln135–136. Intermediate conformations are colored in grey. (**B**) A zoomed-in version of the image is also provided.

**Figure 2:**
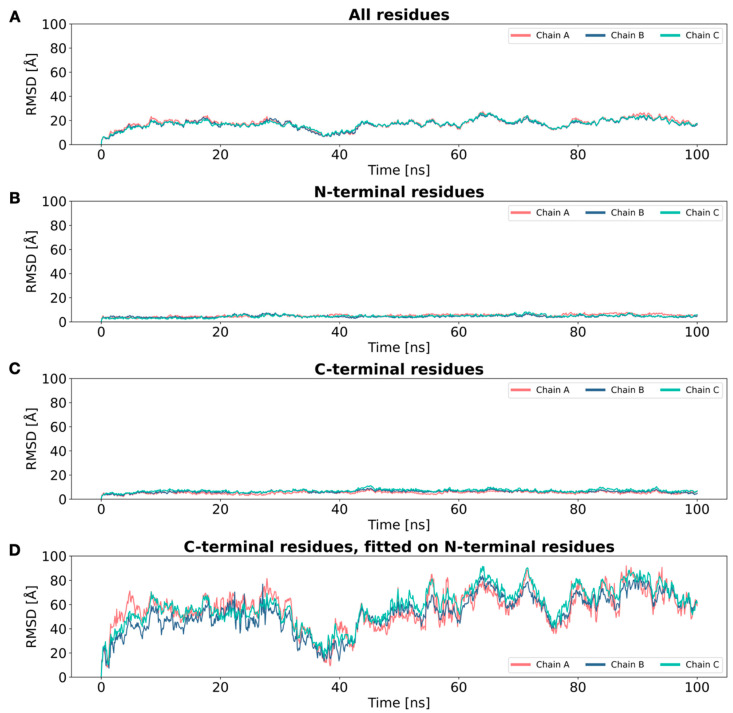
ANGPTL3 RMSD profiles. (**A**) RMSD profile calculated on the overall structure. (**B**) RMSD profile calculated on the N-terminal portion (residues 18–135). (**C**) RMSD profile calculated on the C-terminal portion (residues 136–221). (**D**) RMSD profile calculated on the C-terminal portion following superposition over the N-terminal portion. This series of calculations highlights that the fluctuation of the overall RMSD profile can be attributed to the significant mobility of the final 86 residues subsequent to the bend. For consistency, the four plots have the same scale on the *y* axis. Supplementary Figure S8 allows a better appreciation of the different profiles.

As all the models seemed to behave in a very similar way, the choice of the best one to use for the further steps was not straightforward. However, after evaluating all the data obtained from MD simulations, ANGPTL3 CC 18 was chosen for the further investigations, with reference, first, to cluster analysis data and, second, to its secondary structure features. Starting from an “ideal” coiled-coil conformation, the homotrimer experiences important conformational changes, specifically regarding the orientation of the C-terminal portion of the protein (Figure 1). An in-depth analysis regarding the RMSD profile of the different portions of the trimer, divided by chain, highlighted that, even though the overall RMSD does not seem to reach a plateau, this is not due to structural unfolding but to rearrangements in the 3D structure of the C-terminal region. Specifically, when calculating the RMSD of the first 117 residues and of the last 86 residues separately, before and after a noticeable bend is observed, the RMSD reaches a plateau at relatively low values (~6 Å). On the other hand, when calculating the RMSD of the last 86 residues, after structural superposition on the first 117 residues, the profile noticeably resembles the oscillating profile of the overall RMSD calculation (Figure 2 and Figure S8A–D). The correlation matrix calculated between the RMSD profiles shows the concordant behavior of the chains, as well as a strong relationship between overall RMSD values and C-terminal fluctuations (Supplementary Figure S8E). In conclusion, these results seem to showcase a correct folding of the individual portions, although they exhibit significant movement relative to one another.

### 2.2. Homology Modeling of EL

The homodimeric EL model was obtained via homology modeling, using as a template the experimentally solved structure of LPL (PDB code: 6OAU), a protein functionally similar to EL. The sequence alignment resulted in identity with the query of 49.98%, a similarity of 61.2%, with a coverage of 87%; therefore, a good-quality model was to be expected. The sequence alignment is shown in Supplementary Figure S9.

**Figure 3:**
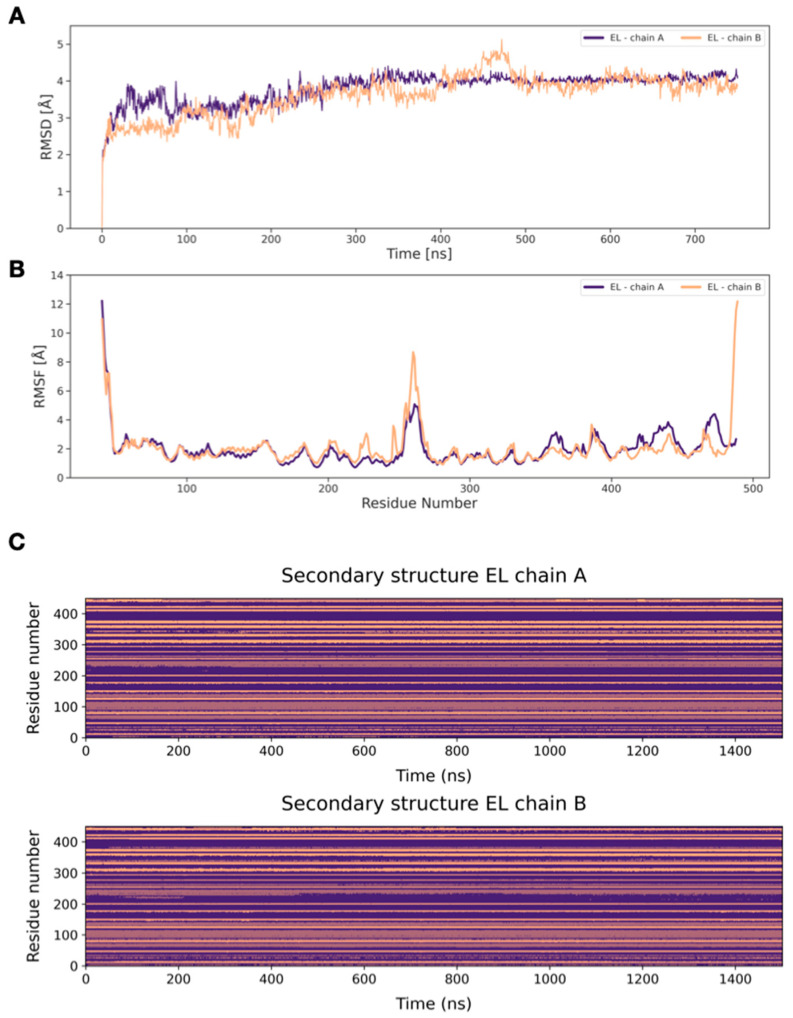
RMSD values of EL. (**A**) RMSD of each of the two chains of EL. The RMSD profiles reach a plateau at relatively low values (~4 Å). (**B**) RMSF profile of each of the two chains of EL. Aside from the N- and C-termini, and the residues connecting the two monomer domains, all residues demonstrate RMSF values < 4 Å. (**C**) Secondary structure of the two EL chains (violet = no secondary structure, light violet = α-helix, salmon = β-sheet), chain A on the top and chain B on the bottom. It clearly shows how both chains maintain the secondary structure throughout the MD simulations.

**Figure 4:**
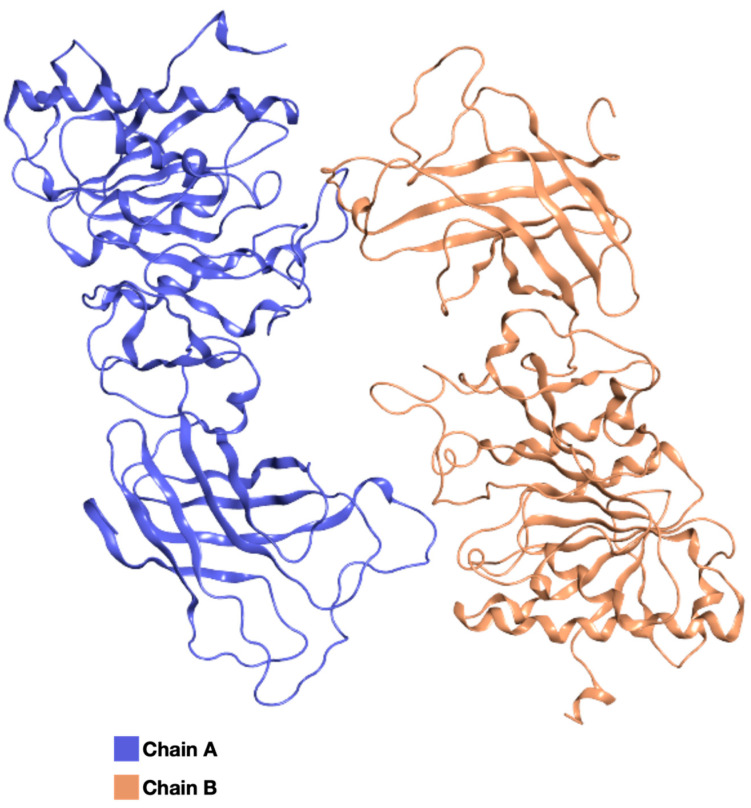
EL most representative structure. Medoid from the most populated cluster of EL MD simulation, corresponding to frame 1163, is represented, colored by chain.

To relax and equilibrate the structure, a MD simulation was carried out on the EL model. The RMSD profile calculated on the individual chains reached a plateau at relatively low values (~4 Å), indicating that the model has reached structural stability (Figure 3A). The RMSF profile shows noticeable mobility at the termini of monomers and in the loops located in the middle, while in the remaining parts of the protein, the fluctuation is ~2 Å (Figure 3B). Figure 3C shows how the secondary structure of the two chains remains constant during the entire MD simulations, a clear indication of structural stability and, conversely, of model quality. Supplementary Figure S10A,B show, respectively, the cluster analysis of the trajectory and the Ramachandran plot of the most populated cluster medoid, whose structure is shown in Figure 4. The results of the cluster analysis further highlight the structural and conformational stability of the model, especially after 300 ns of dynamics.

### 2.3. ANGPTL3∷EL Docking

The medoids of the most populated cluster for both ANGPTL3 and EL were docked. Both MOE and Piper were used for this purpose because they implement different docking algorithms, allowing a more detailed analysis of this interaction. As a result, each program returned 100 ANGPTL3∷EL complexes.

**Figure 5:**
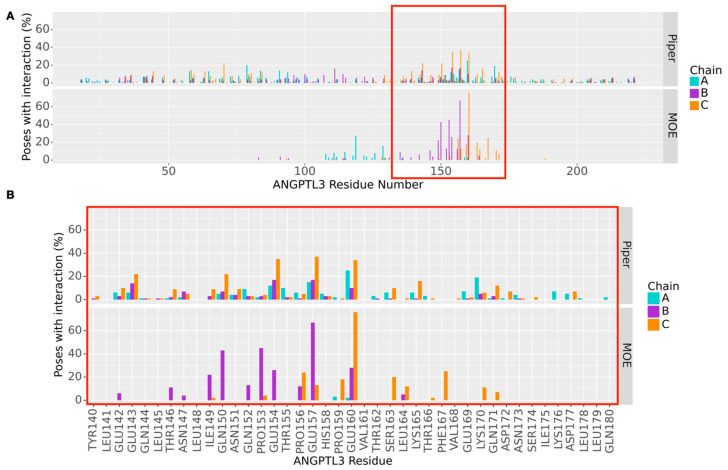
Analysis of protein–protein interactions across docking poses. (**A**) Fingerprint analysis of ANGPTL3-EL interactions in all the docking poses produced by MOE and by Piper. This analysis clearly identifies among the poses a specific portion more involved in interactions. (**B**) Focus on the most interacting portion of ANGPTL3, between Glu143 and Gln171, identified as preferentially involved in the interaction through fingerprint analysis.

Figure 5A shows the fingerprint analysis of all docking poses obtained by MOE and Piper. A clear preference towards a specific portion highly involved in interactions can be observed among the MOE poses. With the Piper poses, instead, the identification of such a portion is not as evident. However, despite the great difference in algorithms and ranking methods, the Piper poses reveal interactions that are twice more present than anywhere else in the same region as MOE, as shown in detail in Figure 5B. It is also important to remember that each Piper pose analyzed must be considered as the medoid of a pose cluster, containing poses similar to the one being showcased. All these results, therefore, help in the identification of a region of recognition by EL on ANGPTL3, between Glu143 and Gln171, that has been conserved in most of the poses and across two different docking software programs.

**Figure 6:**
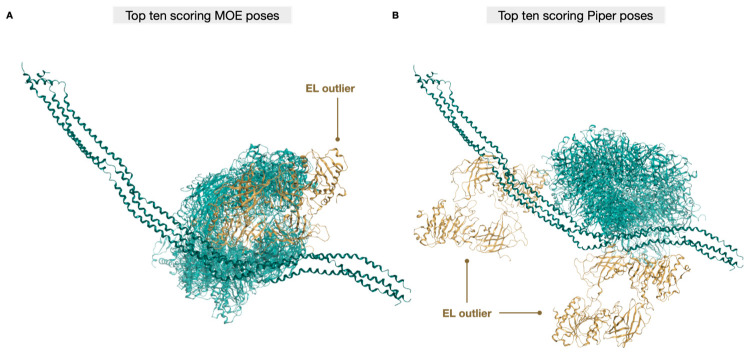
Top ten scoring poses from MOE and from Piper. Best 10 poses obtained by (**A**) MOE and (**B**) Piper. Except for very few exceptions, colored in beige, all the best poses from both programs identify a specific portion and binding mode for ANGPTL3.

The 10 top-scoring poses from MOE and from Piper are shown in Figure 6A and Figure 6B, respectively. Although the two software programs rank the poses with different criteria, EL (cyan) interacts with the same portion of ANGPTL3 (teal) in all the best poses, except one MOE pose and two Piper poses (beige).

Table 1 reports the ranking values for each pose. The cluster size is shown for Piper poses and the S score energy is reported for MOE poses, as they are the respective metrics used to rank the results. It is important to remember that the S scores, while extremely useful to compare poses generated with the same parameters, should not be thought of as absolute energy values.

### 2.4. MD Simulations of Docking Poses and Interactions Analysis

To further confirm the docking results and the interaction modes between ANGPTL3 and EL, three MD simulation replicas were run for 100 ns on four different poses. Three top-scoring poses from MOE (pose 1, 2, and 3) and the medoid of the most populated pose cluster obtained by Piper were selected. Only one pose generated by Piper was studied further via the MD simulation because it is actually representative of a 41-pose cluster, and the second and third clusters were characterized by the same interacting region. 

**Figure 7:**
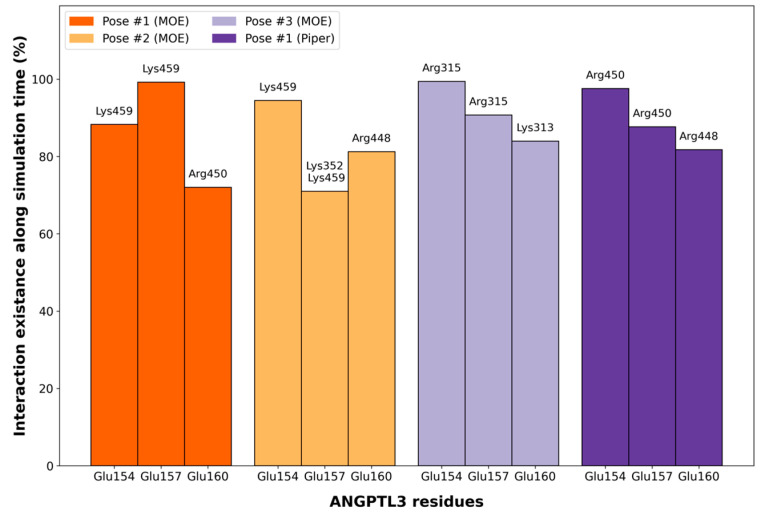
ANGPTL3-EL most persistent interactions during MD simulations of the four poses from protein–protein docking. The residues of EL are reported on top of the bars, while the ones of ANGPTL3 are on the bottom. In each MD, the most persistent interactions involve three specific residues, three glutamates, suggesting a key role in both recognition and interaction.

Figure 7 shows the three residues for each simulation involved in the longest-lasting interactions, while Supplementary Figure S11 reports all the interaction lasting at least 20% of the simulation time. All the longest-lasting interactions of ANGPTL3 with EL involve three ANGPTL3 key residues: Glu154, Glu157, and Glu160.

**Figure 8:**
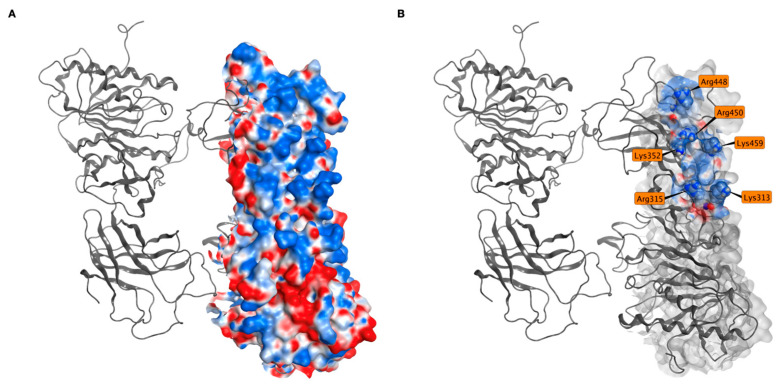
Positive electrostatic surface on EL. (**A**) On the exposed EL surface, there is a large positive (blue) patch and smaller negative (red) and neutral (white) patches (**B**). Residues involved in interactions with ANGPTL3 are in this positive patch.

All these interactions, in fact, are made with only a few EL positively charged residues, namely Lys313, Arg315, Lys352, Arg448, Arg450, and Lys459, all belonging to the same (and the largest) positive patch on the EL exposed surface (Figure 8A,B).

The minimum distances between the key ANGPTL3 glutamates and the respective EL amino acids during MD simulation replicas are shown in Supplementary Figure S12. All the distances are under 5 Å, which corresponds to the threshold for interaction existence. Although there are some differences between replicas, these are in line with the variability of the method, and the data confirm the stability of interactions for most of the MD simulation time.

**Figure 9:**
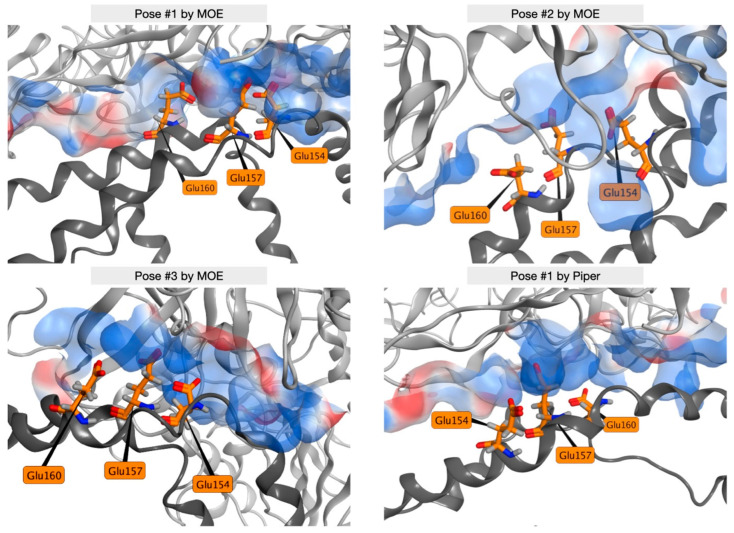
ANGPTL3 key residues recognize a positive electrostatic patch on EL. Positive electrostatic surface (blue) on EL (light gray) in interaction with the three negatively charged glutamic acid residues (orange) of ANGPTL3 (dark gray) in the docking poses used for MD simulations.

Figure 9 shows the key ANGPTL3 residues Glu154, Glu157, and Glu160 interacting with the electrostatic surface of EL in the most representative frame of the MD for each of the selected docking poses. Only the Glu residues in each chain involved in the strongest interactions are shown. During molecular docking calculations, there was no bias towards glutamates of one specific chain of ANGPTL3; all the glutamates are displayed in Supplementary Figure S13. 

## 3. Discussion

ANGPTL3 has been identified as an interesting novel therapeutic target for the treatment of dyslipidemia. The present work investigated the interactions of its N-terminal portion and EL, another important player in the lipoprotein metabolism, in order to understand for the first time the structural basis of its inhibitory activity. In the absence of already experimentally solved structures, three-dimensional models were generated for both proteins, a trimeric coiled coil for ANGPTL3 and a homodimer for EL.

The homology model of EL was built using a reliable template, the experimentally solved structure of homodimeric LPL, a functionally similar protein, which demonstrated a high sequence identity, similarity, and coverage. Therefore, the model could already be considered reliable, but further analysis was performed via MD simulations. The RMSD profile showed that convergence was reached, and the RMSF profile shows noticeable mobility at the termini of monomers and in the loops located in the middle, while in the remaining parts of the protein, the fluctuation is as low as 2 Å. Cluster analysis also highlights a common stable conformation after the first 300 ns of simulation. Globally, all the considered geometrical parameters indicate that the EL model reaches stability in the homodimeric form, the preferred assembly state for the protein according to the available literature [17].

On the contrary, no suitable template was found for the ANGPTL3 N-terminal domain. Therefore, ab initio methodologies had to be employed. Specifically, because of literature data hypothesizing that the protein forms a trimeric coiled coil, CCBuilder was used to build an “ideal” starting point [5]. In order to avoid biases deriving from the starting residues of the α-helices, which would determine the exposed residues, four models were generated and evaluated via MD simulations. No major differences could be recognized among the models, and model CC18 was chosen for further evaluation on the basis of the results of the clusters and the secondary structure analyses. According to the available literature, this is the first reliable model of the trimeric N-terminal of ANGPTL3.

Because of the inherently flawed starting point of the trimeric model and the features of the complex, high values of RMSD were to be expected, as well as the absence of a plateau, if considering the protein as a whole. However, upon segmenting the RMSD calculation before and after the bend, it was possible to observe that the pronounced fluctuations in the overall RMSD derive from substantial motion exhibited by the final 86 residues relative to the first 117, rather than being indicative of structural instability. Cluster analysis highlights that the trimer mainly explores two conformations, in a dynamic equilibrium, as shown in Figure 1. 

Since no information about the EL-ANGPTL3 interaction is available, two different protein–protein docking softwares were used to generate the heteromeric complex. The results identify a specific ANGPTL3 portion as the most probable one for the interaction. However, its identification in the Piper poses is not as evident as with MOE, if only the medoid for each Piper cluster in considered. This is an algorithm-dependent effect that was expected and in line with the ranking algorithm used by Piper. In fact, the ranking of the poses in Piper is based on geometrical clustering, a process that provides a more spatially distributed set of poses. On the contrary, the MOE ranking algorithm gives priority to energetically favorable regions. Interestingly, the S scores of the best MOE poses were very similar. This suggests that slight position variations do not significantly impact the affinity between the two proteins. Despite the differences between the two algorithms, the results were largely in agreement, suggesting a key role in the interaction with EL of three negatively charged ANGPTL3 residues, namely Glu154, Glu157, and Glu160. On EL, a patch of positively charged exposed surface appeared consistently as the interaction region. All the investigated interactions proved to be stable during 100 ns MD simulations replicas, with a permanence of at least 70%. Interestingly, the positive residues on the surface of the EL were not always the same, but this did not affect the stability of the interactions.

## 4. Materials and Methods

### 4.1. De Novo Modeling of the N-Terminal ANGPTL3 Trimer

To model the 3D structure of the N-terminal domain of ANGPTL3, a protein BLAST [18] search was performed to find homologues, using the sequence downloaded from UniProtKB [19]. Since no suitable templates were found due to the lack of coverage on the N-terminal domain, a preliminary sequence-based secondary structure prediction (SSP) was performed with PsiPred 4.0 (Bioinformatics Group, Department of Computer Science, University College London, Gower Street, London WC1E 6BT, UK) [20], which incorporates two feed-forward neural networks performing an analysis on the PSI-BLAST output. Then, the N-terminal domain of mature ANGPTL3, without the signal peptide and cleaved at Arg221, was modeled as an ideal coiled coil using CCBuilder 2.0 (School of Chemistry, University of Bristol, Cantock’s Close, Bristol, BS8 1TS, UK) [21]. Using the amino acids of an input sequence, this tool can build 3D structures containing a backbone with ϕ and ψ dihedral angles forming “ideal” coiled α-helices using the amino acids of an input sequence, therefore requiring very careful subsequent structure equilibration steps. Default parameters were used for radius, pitch, and interface angles. Since no experimental data were available about the correct winding of the coiled coil, considering that α-helices contain ~4 amino acids per turn, four different models of the N-terminal domain of ANGPTL3 were generated, starting from 4 consecutive amino acids: Ser17 (ANGPTL3 CC 17), Arg18 (ANGPTL3 CC 18), Ile19 (ANGPTL3 CC 19), Asp20 (ANGPTL3 CC 20). The four proposed models were submitted to MD equilibration, clustering, and secondary structure analysis to obtain the best candidate for further investigation.

### 4.2. Homology Modeling of the Endothelial Lipase Dimer

The 3D structure of EL was modeled with Prime (Prime, Schrödinger, LLC, New York, NY, USA, 2021) using a homology modeling procedure. A protein BLAST search was performed to find homologues to be used as suitable templates, using BLOSUM80 as the substitution matrix. The chain A of the apo structure of wild-type LPL (PDB ID: 6OAU [22]) was identified as a suitable template, and its structure was downloaded from the RCSB PDB database [23]. After aligning the sequences, an EL monomeric tridimensional model was obtained, using the knowledge-based method, which builds insertions and closes gaps using segments from known structures. Then, to build the homodimeric model of EL, the monomer was duplicated and, starting from the homodimeric crystal of LPL, each chain of EL was superposed to each chain of LPL, correctly orienting the homodimer in 3D. Next, the 3D homology model was prepared for molecular dynamics simulation by adding missing hydrogens, optimizing H-bond assignments, and, in the end, performing a restrained minimization on the 3D structure.

### 4.3. MD Simulations

All the systems for MD simulations were prepared using the Desmond [24] System Builder tool. The system was solvated with water molecules parametrized using the SPC model. The box was built to fit the whole protein, plus a buffer to account for protein movements (10 Å for EL system and 15 Å for ANGPTL3 system). Chloride and sodium ions were added to neutralize the system and reach a concentration of 0.15 M [25,26]. Supplementary Table S1 reports the size of the boxes and the respective number of atoms. MD simulations were performed using Desmond [27] using the following protocol for the equilibration step:

1. NPT ensemble, 12 ps at 10 K 10 K and restraints of 50 kcal/mol/Å on protein atoms;
2. NVT ensemble, 12 ps at 10 K and restraints of 50 kcal/mol/Å on protein heavy atoms;
3. NPT ensemble, 12 ps at 10 K and restraints of 50 kcal/mol/Å on protein heavy atoms;
4. NPT ensemble, 12 ps at 300 K and restraints of 50 kcal/mol/Å on protein heavy atoms;
5. NPT ensemble, 24 ps at 300 K without restraints.

The following parameters were used for the production stage: OPLS4 force field [28], periodic boundary conditions (PBC), temperature 300 K with Nose-Hoover thermostat, pressure 1 bar coupled with a Martyna-Tobias-Klein piston, integration step 2 fs, saving a frame every 0.5 ps. 

The EL model was run for 750 ns to reach an RMSD plateau. On the other hand, the simulations for the ANGPTL3 models were run only for 100 ns, because of the size of the systems (see Supplementary Table S1). 

### 4.4. MD Analysis

For each trajectory of ANGPTL3 and EL, the RMSD and the RMSF were calculated on α carbons using the Schrödinger API. Cluster analyses were also performed according to the GROMOS [29] method, using as the cluster distance the RMSD matrix of α carbons with respect to the first frame of the MD simulation [30]. The threshold was set to 7 Å for ANGPTL3 and to 2.1 Å for EL. ANGPTL3 trajectories underwent a further evaluation of the secondary structure content; along each MD frame, the percentage of time spent in an α-helix compared to the total simulated time was evaluated residue by residue. The correlation matrix was computed using R. 

### 4.5. Protein–Protein Docking

The MOE 2020.02 (Montreal, QC, Canada) Protein–Protein Dock module [31,32] and the PIPER FFT-based protein–protein docking program (Schrödinger Suite 2021-4 [33]) were used to dock EL on ANGPTL3 [32]. Protein–protein docking was performed with two different algorithms in order to cross-validate our results and provide further robustness to the data. Briefly, the protein–protein docker of MOE uses a multi-stage method for generating poses and then ranking them. Starting from a coarse-grained (CG) model to reduce the computational search space, exhaustive sampling is carried out to generate a set of initial poses. A set of uniformly distributed rotations is generated [31] and a Fast Fourier Transform (FFT) is used to sample all translations for each rotation. The final ranking is based on the GBVI/WSA DG, a force field-based scoring function that estimates the free energy of the binding of the ligand from a given pose. Conversely, the Piper docking algorithm starts with a rigid global search based on the FTT correlation approach. The retained structures are clustered using the pairwise RMSD as the distance measure and a fixed or variable clustering radius. The structures in these clusters are refined by a novel medium-range optimization method which was developed to locate the global energy minima within the regions of the conformational space defined by the separate clusters.

The medoid, a representative structure with the minimum distance from the others, of the most populated cluster of the ANGPTL3 MD simulation was set as the receptor, while the medoid of the most populated cluster of the EL MD simulation, given its more compact structure, was set as the ligand. Every program generated 100 poses, ranked by cluster size in Piper and by score energy in MOE.

The interactions of all the poses were globally evaluated with the MOE Protein–Ligand Interaction Fingerprint (PLIF) module for MOE docking poses and with the Schrödinger Interaction Fingerprints tool for Piper docking poses, filtering for hydrogen bonds and charged contacts.

To assess the interactions and the stability of the complexes, three 100 ns replicas with the parameters described earlier were run for each of the three top-scoring docking poses obtained by MOE and for the first top-scoring pose obtained by Schrödinger. The interactions between ANGPTL3 and EL during the simulations originating from docking poses were calculated with an in-house script [34] and evaluated in terms of occupancy in 100 ns of the MD simulation (mean of the three replicates).

The minimum distance along the trajectories between acceptor and donor atoms of the best ANGPTL3-EL interactions was computed with CPPTRAJ [35].

## 5. Conclusions

The role of the inhibitory effect on EL by ANGPTL3 has gained attention in the last few years, as soon as its relevance in the lipoprotein metabolism became evident. However, no experimental evidence was available on the exact mechanism of this inhibition. This study provides for the first time a 3D model of the N-terminal ANGPTL3 trimer in its coiled-coil conformation. Molecular dynamics simulations were used to equilibrate the trimer and characterize its behavior in its physiological environment. This equilibrated model was used to characterize ANGPTL3 interactions with EL at an atomistic level, using both molecular docking and molecular dynamics simulations of the resulting complexes. The data obtained with different but concordant algorithms supports the hypothesis of a direct inhibition mechanism. In particular, three ANGPTL3 glutamates were predicted to interact with a positively charged patch on the surface of the EL.

The in silico evidence generated in this work provides a solid hypothesis that could guide the design of further in vitro experiments studying the interaction between ANGPTL3 and EL. It is, in fact, important to elucidate this interaction and to identify a binding site that can be a promising target for the development of new drugs for the treatment of dyslipidemias.

## Figures and Tables

**Table 1:** Ranking values of the top ten scoring poses. Ranking values for each pose. For Piper poses, the cluster size is reported. For MOE poses, the S score is reported. The outlier poses are highlighted (* for MOE poses, # for Piper poses).

Pose Rank	Cluster Size (PIPER)	S Score [kcal/mol] (MOE)
1	41	−79.3
2	35	−76.3
3	33	−75.9
4 #	26	−75.4
5 *	26	−71.5
6	25	−70.1
7	24	−68.4
8	24	−68.3
9	23	−68.1
10 #	21	−67.8

## Data Availability

The original contributions presented in the study are included in the article/Supplementary Materials, further inquiries can be directed to the corresponding author/s.

## Supplementary Materials

The following supporting information can be downloaded at: https://www.mdpi.com/article/10.3390/ijms25063555/s1.
