# Supplementary material for: In Silico Description of the Direct Inhibition Mechanism of Endothelial Lipase by ANGPTL3
Source: Int J Mol Sci. 2024 Mar 21;25(6):3555. doi: 10.3390/ijms25063555 (PMC10971391; doi:10.3390/ijms25063555)
Supplement: Supplementary file 1 [file ijms-25-03555-s001.zip › ijms-2885398-supplementary.pdf]

Title:

In silico description of the direct inhibition mechanism of endothelial lipase by ANGPTL3

Linda Montavoci[1,2,†], Omar Ben Mariem[1,†], Simona Saporiti[3], Tommaso Laurenzi[1], Luca Palazzolo[1], Alice Ossoli[4], Uliano Guerrini[1], Laura Calabresi[4], and Ivano Eberini[5,*]

[1] Dipartimento di Scienze Farmacologiche e Biomolecolari, Università degli Studi di Milano, Milan, Italy

[2] Dipartimento di Scienze della Salute, Università degli Studi di Milano, Milan, Italy

[3] Analytical Excellence and Program Management, Merck Serono S.p.A., Rome, Italy

[4] Centro E. Grossi Paoletti, Dipartimento di Scienze Farmacologiche e Biomolecolari, Università degli Studi di Milano, Milan, Italy

[5] Dipartimento di Scienze Farmacologiche e Biomolecolari & DSRC, Università degli Studi di Milano, Milan, Italy

† These authors contributed equally

* Corresponding author: ivano.eberini@unimi.it, via Giuseppe Balzaretti, 9 – 20133 - Milan, Italy

SUPPLEMENTARY MATERIAL
Figures

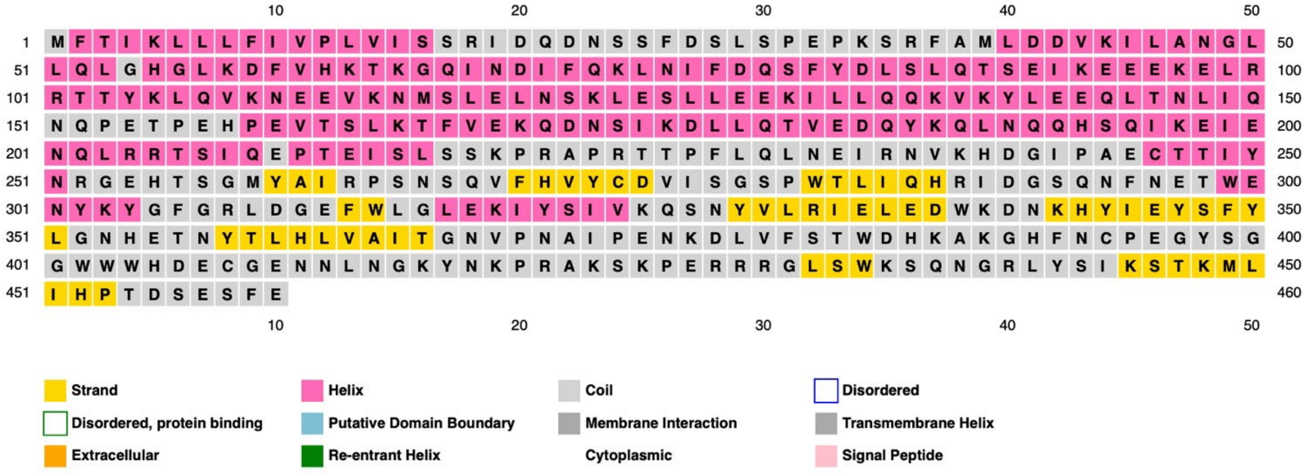

Supplementary figure 1: Secondary structure prediction of ANGPTL3

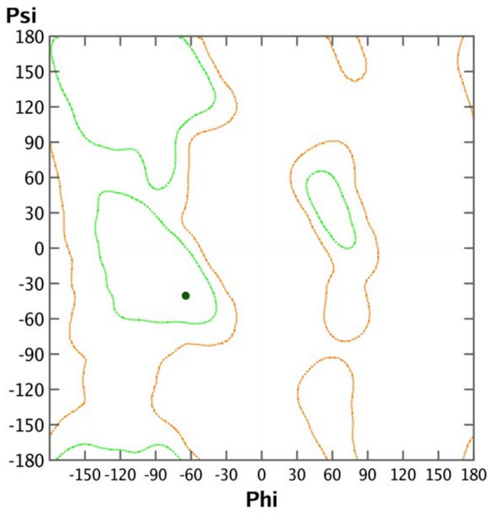

Supplementary figure 2: Ramachandran Plot of ANGPTL3 models obtained with CCBuilder before MD simulations. The phi-psi values for all residues are ideal and identical, resulting in a plot apparently containing one single point.

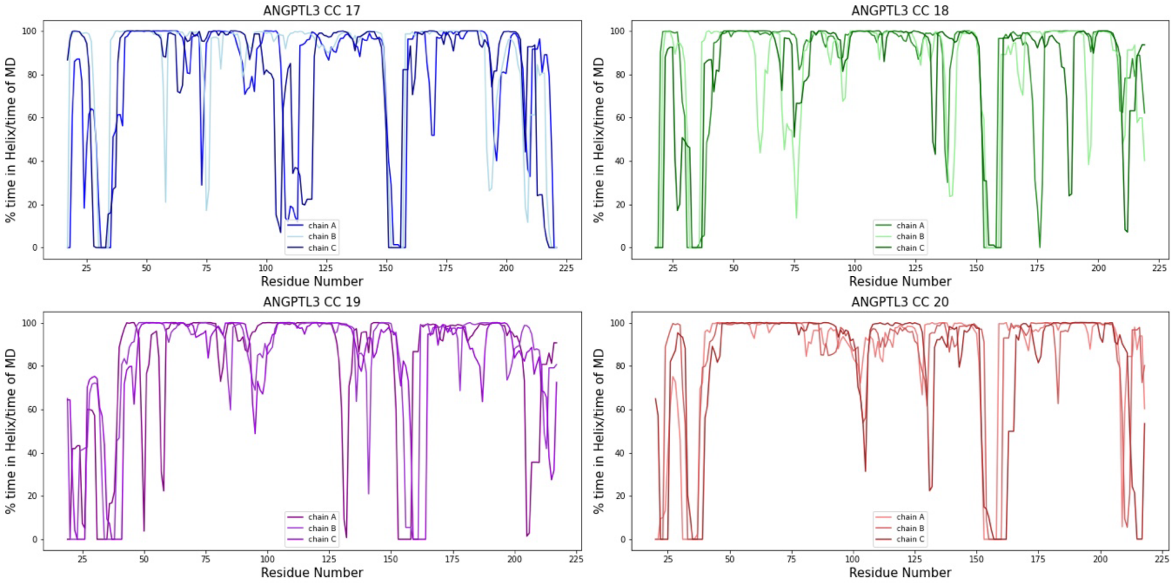

Supplementary figure 3: Time percentage in α-helix of each residue of ANGPTL3 models during MD simulations.

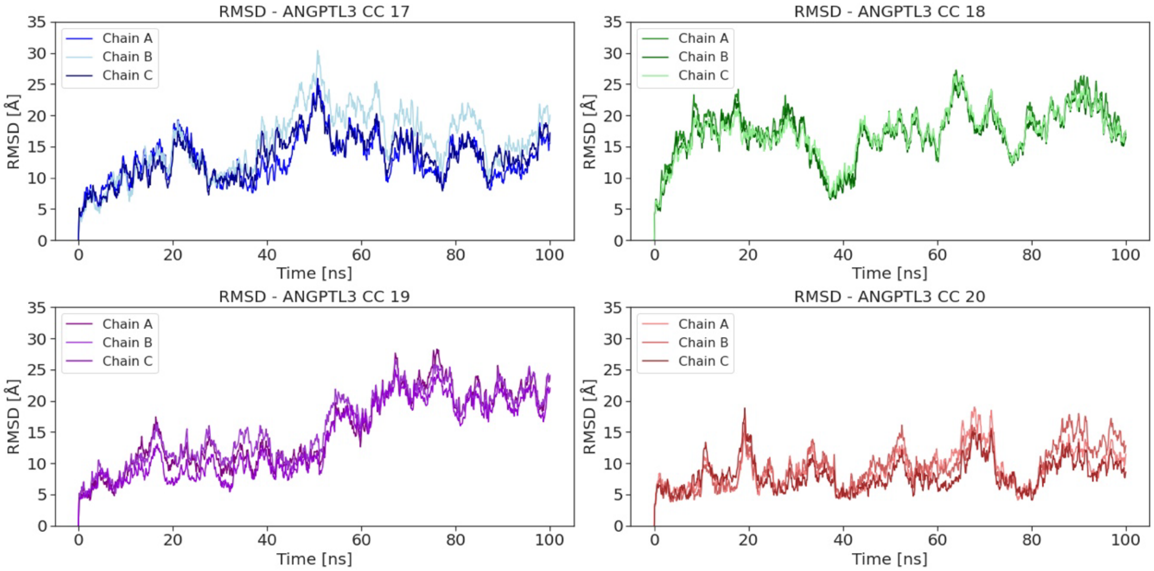

Supplementary figure 4: RMSD during MD simulation of ANGPTL3 models.

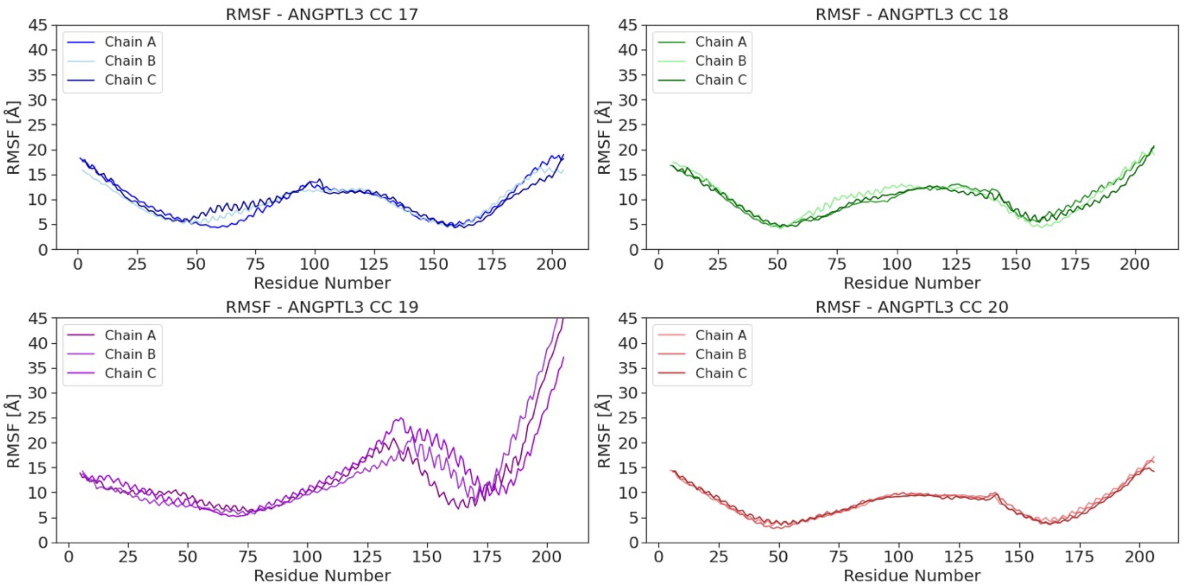

Supplementary figure 5: RMSF during MD simulation of ANGPTL3 models

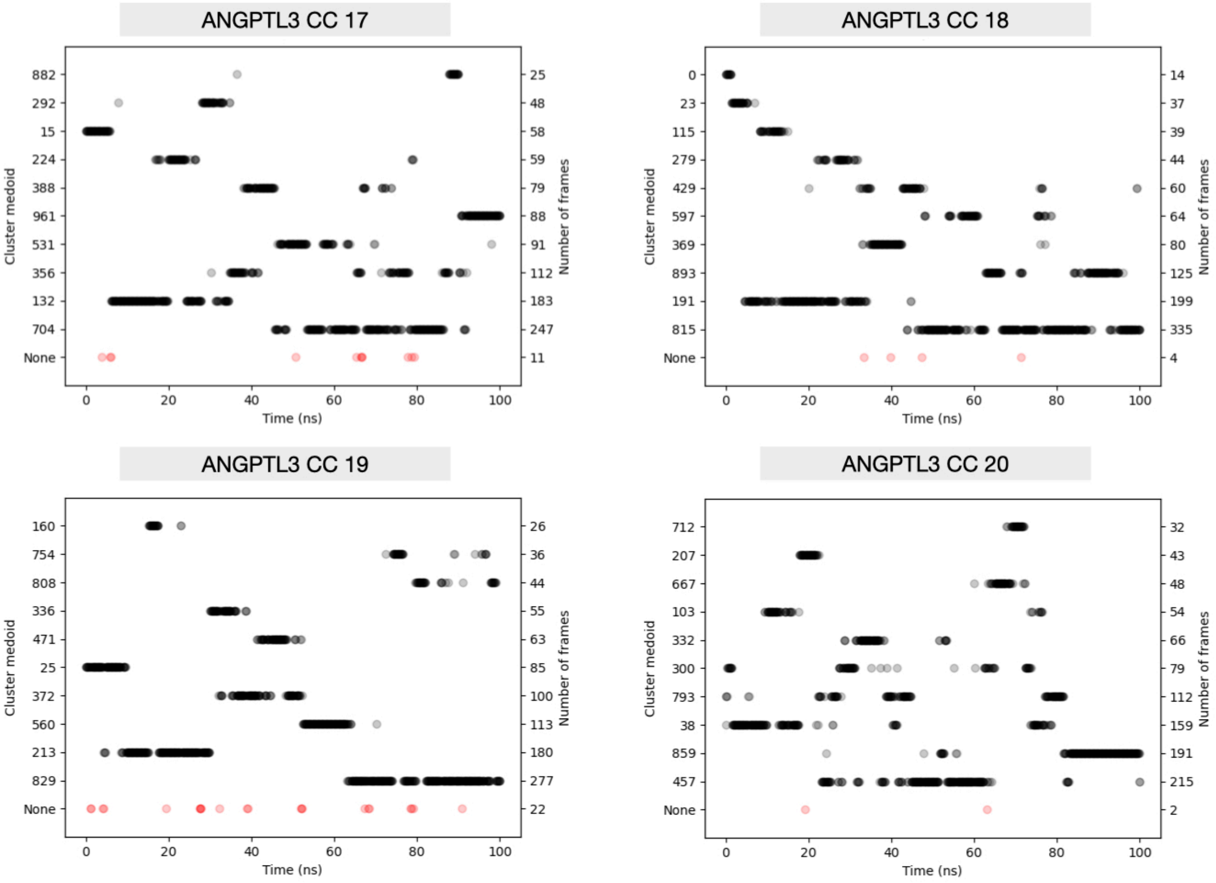

Supplementary figure 6: Cluster analysis performed during MD simulations.

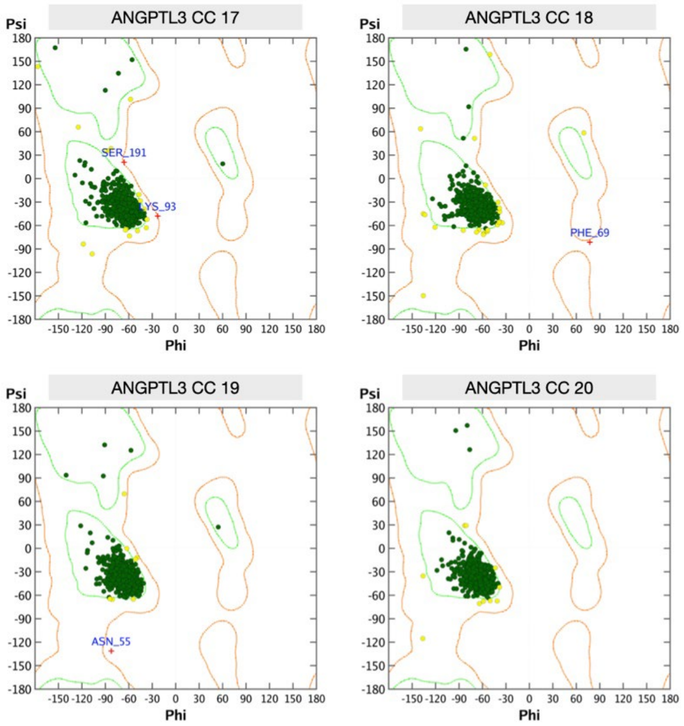

Supplementary figure 7: Ramachandran Plot of ANGPTL3 medoids from the most populated clusters after MD simulation

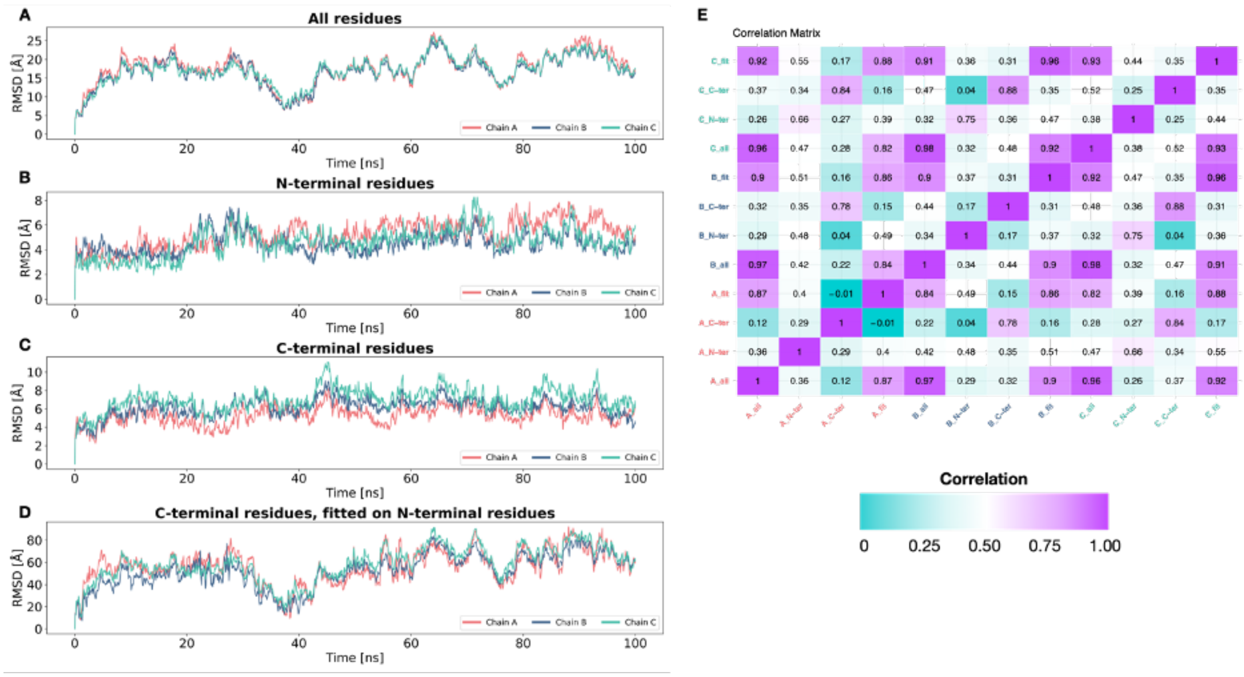

Supplementary figure 8: ANGPTL3 RMSD profiles, referring to Fig. 2, with more appropriate scales on the y-axis for each profile. A) RMSD profile calculated on the overall structure. B)

RMSD profile calculated on the N-terminal portion (residues 18-135). C) RMSD profile calculated on the C-terminal portion (residues 136-221). D) RMSD profile calculated on the C-terminal portion fol-lowing the superposition over the N-terminal portion. This series of calculations highlights that the fluctuation of overall RMSD profile can be attributed to the significant mobility of the final 86 residues subsequent to the bend. E) Correlation matrix calculated between RMSD profiles reported in Fig. 2. It is possible to appreciate the concordant behavior of the chains as well as the strong relationship between overall RMSD values and C-terminal fluctuations.

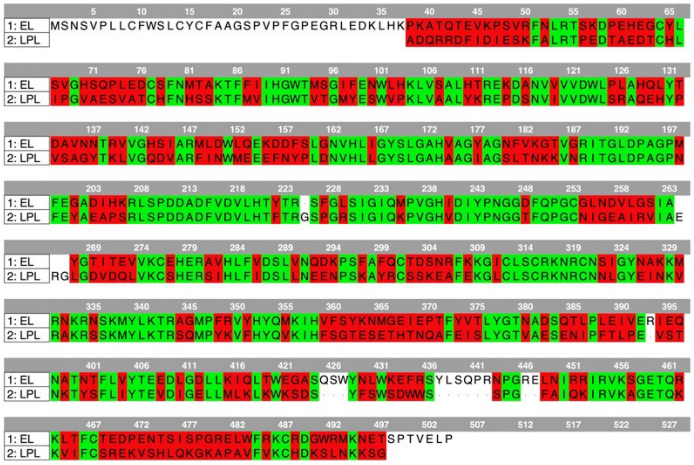

Supplementary figure 9: Alignment between query sequence of EL and template sequence of LPL. The residues are colored by identity, where red indicates different residues and green means same residues.

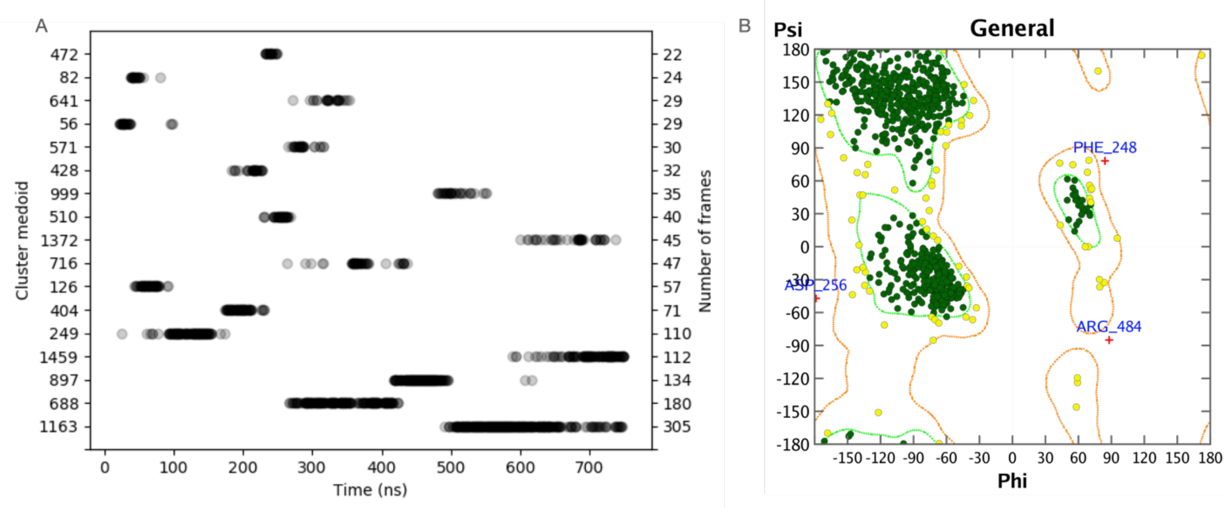

Supplementary figure 10: Cluster analysis (A) and Ramachandran Plot (B) of MD simulation of EL

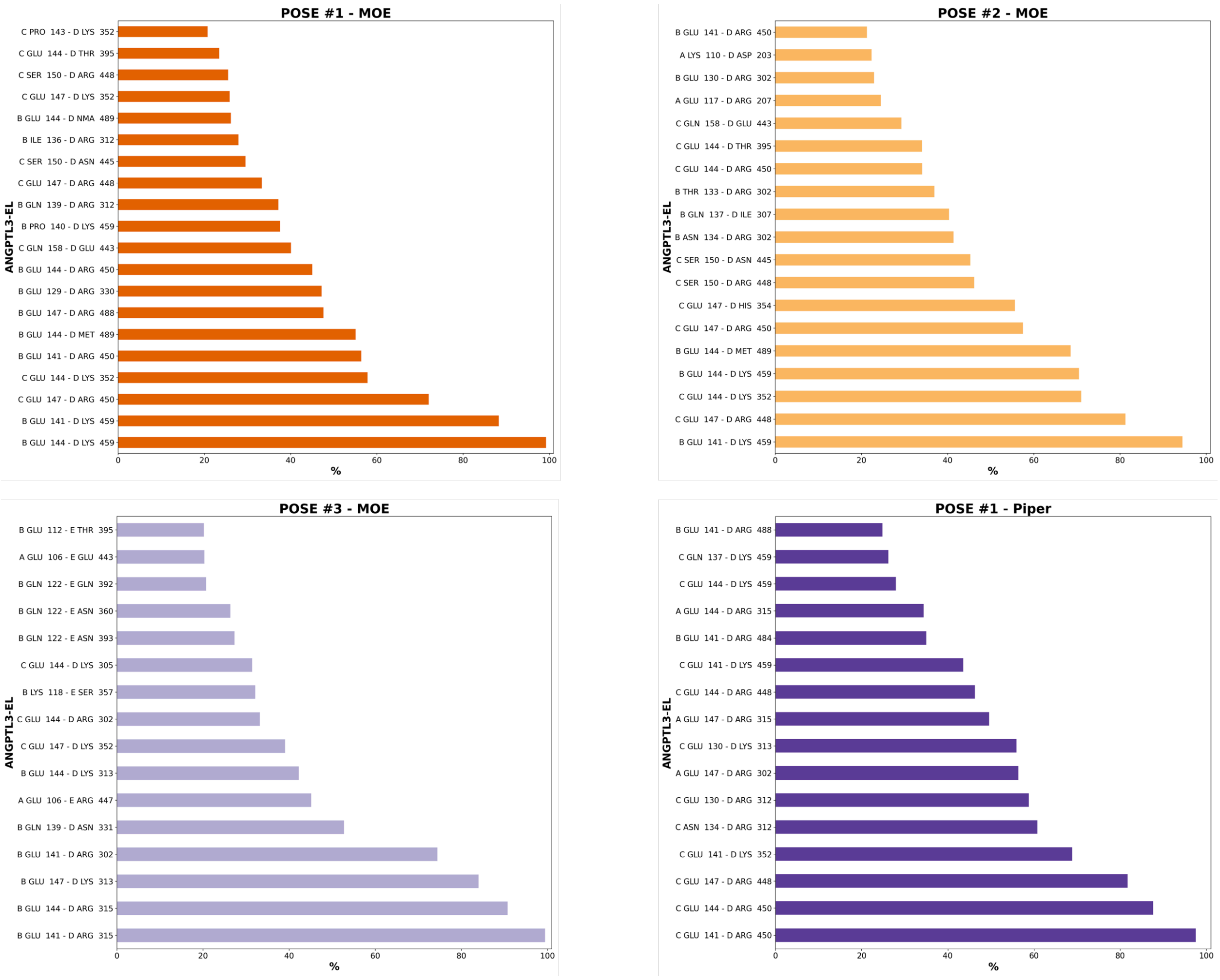

Supplementary figure 11: Average occupancy of all the interactions during MD simulation of the four poses.

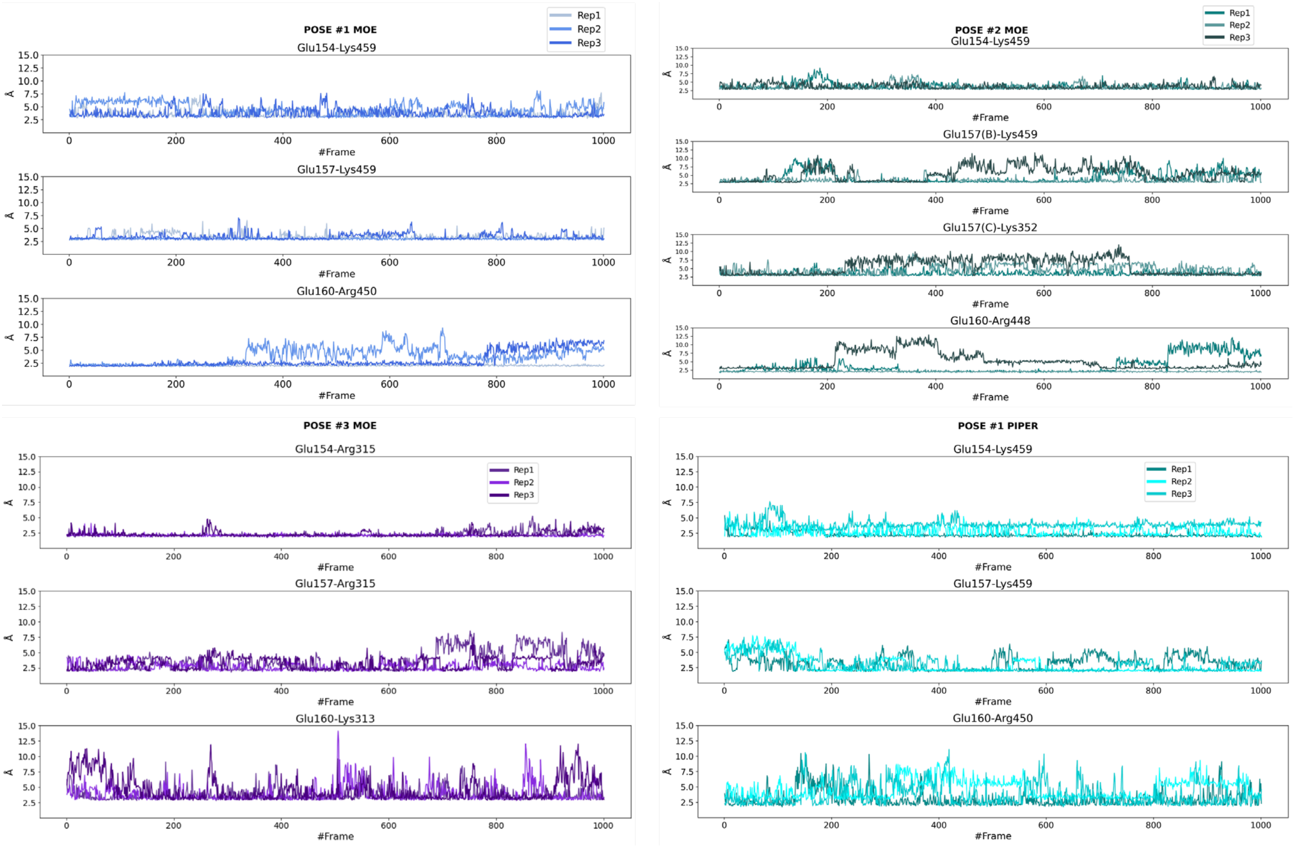

Supplementary figure 12: The minimum distances between ANGPTL3 key residues and the corresponding EL residues are under 5 Å threshold and confirm the presence of interactions for the most simulation time.

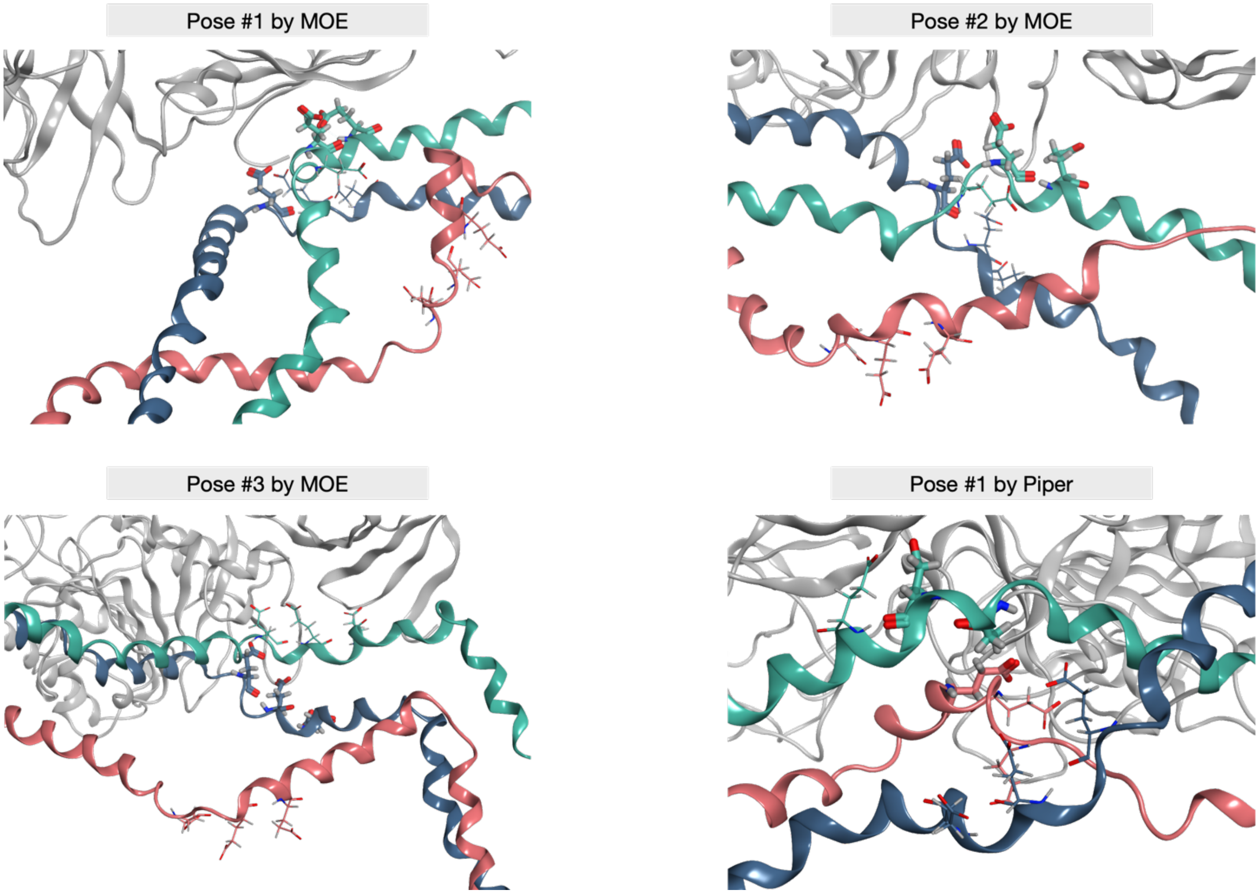

Supplementary figure 13: All Glu154, Glu157 and Glu160 are displayed and colored by chain. The interacting glutamates are bolded. EL is colored in gray.

Simulation	Box sizes (Å)	# of atoms
EL	98x152x76	112160
ANGPTL3 CC 17	149x119x327	567970
ANGPTL3 CC 18	149x119x327	568223
ANGPTL3 CC 19	149x119x327	568067
ANGPTL3 CC 20	149x119x327	568109
EL::ANGPTL3 MOE-1	108x145x314	484744
EL::ANGPTL3 MOE-2	108x145x314	483698
EL::ANGPTL3 MOE-3	108x144x314	484534
EL::ANGPTL3 PIPER	116x134x318	484845

Supplementary table S1: Box sizes and number of atoms for all MD simulations described.
